# Molecular Mechanisms Related with Oligometastatic Prostate Cancer—Is It Just a Matter of Numbers?

**DOI:** 10.3390/cancers14030766

**Published:** 2022-02-01

**Authors:** Cristian Surcel, Alexander Kretschmer, Cristian Mirvald, Ioanel Sinescu, Isabel Heidegger, Igor Tsaur

**Affiliations:** 1 Center of Urologic Surgery, Dialysis and Renal Transplantation, Fundeni Clinical Institute, “Carol Davila” University of Medicine and Pharmacy, 00238 Bucharest, Romania; cmirvald@gmail.com (C.M.); urologiefundeni@gmail.com (I.S.); 2 Department of Urology, Ludwig-Maximilians University, 81377 Munich, Germany; Alexander.Kretschmer@med.uni-muenchen.de; 3 Department of Urology, Medical University Innsbruck, 6020 Innsbruck, Austria; isabel-maria.heidegger@i-med.ac.at; 4 Department of Urology and Pediatric Urology, University Medical Center Mainz, 55131 Mainz, Germany; Prof.Dr.med.Igor.Tsaur@unimedizin-mainz.de

**Keywords:** oligometastatic prostate cancer, biomarkers, PET CT, genes, microRNA

## Abstract

**Simple Summary:**

Oligometastatic prostate cancer represents a transitional state between localized and widespread metastatic disease and it is defined by presence of five or fewer metastatic sites. In the current narrative review, we provide an overview of the current treatment landscape of oligometastatic cancer, focusing on the current biomarkers used in the identification of true oligometastatic disease and highlighting the impact of molecular imaging on stage shift in different scenarios. Finally, we address current and future directions regarding the use of genetic and epigenetic targeting treatments in oligometastatic prostate cancer.

**Abstract:**

During the last decade, the body of knowledge regarding the oligometastatic state has increased exponentially. Several molecular frameworks have been established, aiding our understanding of metastatic spread caused by genetically unstable cells that adapt to a tissue environment which is distant from the primary tumor. In the current narrative review, we provide an overview of the current treatment landscape of oligometastatic cancer, focusing on the current biomarkers used in the identification of true oligometastatic disease and highlighting the impact of molecular imaging on stage shift in different scenarios. Finally, we address current and future directions regarding the use of genetic and epigenetic targeting treatments in oligometastatic prostate cancer.

## 1. Introduction

During the last decade, the body of knowledge regarding the oligometastatic state has increased exponentially. Several molecular frameworks have been established that aid the understanding of metastatic spread caused by genetically unstable cells that adapt to a tissue environment which is distant from the primary tumor [1]. Genomic biomarkers are crucially required in order to discriminate between indolent or aggressive disease and provide data to guide the treatment decision. Hereby, genetic-, epigenetic- and immune-related pathways have been described [2] and will be further elaborated in this review.

The concept of oligometastatic disease as a transitional state between localized disease and diffused metastatic spread, as proposed by Hellmann and Weichselbaum in 1995, is still under debate [3]. However, clinical adaptation is ongoing; for colorectal cancer, it has been shown that patients with a limited number of liver and, to a lesser extent, pulmonary metastases, can have excellent long-term outcomes if metastasis-directed therapy strategies are applied. Nevertheless, the concept is not universally accepted and supporting evidence is still mainly based on retrospective studies [4].

The definition of oligometastasis in prostate cancer is usually based on the number of metastases, and there is no generally accepted definition of oligometastatic prostate cancer (omPCa) to date. Most clinical trials have included patients with a maximum of three to five metastases.

The current literature describes several subgroups of omPCa: (1) de novo (synchronous) oligometastatic prostate cancer; (2) oligorecurrent (metachronous) prostate cancer, defined as a metastatic recurrence following previous definitive therapy of the primary tumor; and (3) oligoprogressive prostate cancer, defined as a progression of a small number of metastases in otherwise stable disease stages [5]. 

The definition of omPCa is usually based on the existence of bone or lymph node metastases. However, to date, it is still to be elaborated whether certain metastatic sites act as checkpoints and prevent further metastatic spread if metastasis-directed therapy is applied. Again, these reflections are supported by the fact that in colorectal cancer, differences in oncological outcomes based on the metastasis site have been described before [4].

In the current narrative review, we provide an overview of the molecular landscape of omPCa, evaluate current biomarkers used in the identification of true oligometastatic disease and highlight the impact of molecular imaging on stage shift in different scenarios. Finally, we address current and future directions regarding the use of genetic and epigenetic targeting treatments in omPCa.

## 2. Biology of Oligometastatic Disease

More than 25 years ago, Hellman and Weichselbaum described, for the first time, the term “oligometastases”, suggesting that in some patients with a limited number of metastatic tumors, the extent of disease exists in a transitional state between localized and widespread systemic disease. It is important to understand that metastases can either arise from the primary tumor (synchronous seeding) or from other metastatic sites (metachronous seeding) [3].

From a biological point of view, metastasis formation initiates with a loss in cellular adhesion, followed by increased motility that allows the primary tumor to enter into the circulation and to metastasize into new organs. Despite intensive research in this area, the exact molecular mechanism of this multistep process is still under evaluation. In general, patients with oligometastatic disease have typically less biologically aggressive tumors, whose metastatic potential is more limited and presents slow-growing features [6]. 

In contrast to other tumor entities such as lung or breast cancer—where it has been demonstrated that oligometastatic lesions originate form malignant cell clones with distinct genetic alterations compared to those causing polymetastatic disease—in prostate cancer, large-scale analyses across solid tumor metastases suggest that most metastatic lesions present subclonal homogeneity [7]. In particular, analyses of treatment-naive metastases have demonstrated that the majority of driver-gene alterations are shared between different metastatic sites [8]. Recent technical advances, such as single-cell sequencing, will provide more insight regarding the oligometastatic state in prostate cancer as there is an urgent need to identify the clonal source of metastatic disease in primary prostate in order to provide personalized patient treatment.

There is some biological evidence supporting the therapeutical concept of metastasis-directed therapy (Table 1). Using whole-genome sequencing, Gundem et al. analyzed metastases in ten patients with metastatic prostate cancer and found metastasis-to-metastasis spread to be a common phenomenon, which occurs either via de novo monoclonal seeding or, less frequently, through the transfer of tumor clones between the respective metastatic sites [9]. In addition, Hong et al. longitudinally analyzed the collected samples of primary tumor and metastatic lesions of four patients with prostate cancer. Hereby, the authors used whole-genome and ultra-deep targeted sequencing. Interestingly, they were able to detect primary as well as metastatic clones even years after the removal of the prostate, and observed inter-metastatic seeding in one in four cases [10].

The evaluation of somatic mutational profiles revealed a spectrum of metastatic biology that helps in redefining oligometastasis. Recently, Deek et al. characterized the somatic mutational landscape across the disease spectrum of metastatic castration-sensitive prostate cancer (mCSPC) to elucidate a biological definition of oligometastatic castration-sensitive PC (defined as ≤5 lesions). The occurrence of driver mutations in TP53, WNT and cell cycle genes increased across the mCSPC spectrum. The TP53 mutation was associated with shorter rPFS and time to a castrate-resistant state in oligometastatic patients [17].

Moreover, a recent prospectively-conducted trial aimed to describe the pathologic characteristics (KI-67 index ≥ 5%, positive PGP 9.5, chromogranin A and synaptophysin) of patients with omPCa initially treated with radical prostatectomy. The authors concluded that oligometastatic prostate adenocarcinoma does not have a specific clinical-pathologic profile [18].

Some studies also suggest that oligometastatic progression can be regulated by epigenetic alterations. Frequently addressed epigenetic alterations in prostate cancer encompass histone modifications, nucleosome remodeling and DNA methylation. However, although these alterations influence patterns of gene expression, they do not result in meaningful changes in the genetic code [19]. 

Recently, there have been several attempts to develop epigenetic targeted therapies in advanced prostate cancer. Variations in DNA methylation are frequently observed in mCRPC disease stages and encompass promoter hypermethylation [20], global hypomethylation [21] and promoter hypomethylation with consecutive activation of proto-oncogenes [22,23]. Since DNA methylation is regulated by DNA methyltransferases (DNMT), the effect of DNMT inhibition has been assessed in patients with prostate cancer. In a phase I/II trial of the DNMT inhibitor azacitidine, Singal et al. assessed a total number of 15 (phase I) and 7 (phase II) patients with mCRPC who progressed during or within 6 months of docetaxel chemotherapy. The authors observed a PSA response >50% in 10 of 19 evaluable patients and an objective radiographic response in 3 out of 10 evaluable patients. However, grade 4 neutropenia was frequently observed and dose reductions were necessary during phase II of the trial [13]. 

Modifications and aberrations of histone complexes have been observed in patients with mCRPC [24]. Hereby, histone deacetylases (HDACs) represent a potentially druggable target of interest, since they are involved in the transcription of androgen receptor-targeted genes [25]. In a recent phase II study, Ferrari et al. assessed the antitumor activity of the HDAC inhibitor panobinostat in 55 patients that were randomized to panobinostat 40 mg or 20 mg in combination with bicalutamide 50 mg/day in 3-week cycles. The authors observed a prolonged radiographic progression-free survival (rPFS) at 36 weeks versus a historic high-dose bicalutamide regimen [11]. 

Recently, the results of a phase I/II trial investigating the bromodomain extraterminal inhibitor ZEN-3694 in combination with enzalutamide were published. Seventy-five patients with progressive mCRPC and previous therapy with next-generation ADT (abiraterone, enzalutamide) were enrolled. The median rPFS was 9 months and composite median radiographic or clinical progression-free survival (PFS) was 5.5 months with a median therapy duration of 3.5 months. Long-term therapeutic effects without detectable progression were observed in 17% (≥12 months) and 5% (≥24 months) of the patients. Notably, more favorable results were found for patients with lower AR transcriptional activity in baseline tumor biopsies as well as for patients with aggressive variant clinical features [15]. It has to be emphasized that none of the current evidence on epigenetic therapies in prostate cancer has been stratified regarding metastases sites and volume. 

It is well known that 30–50% of metastases demonstrate clonal/subclonal heterogeneity [9]. Preliminary genomic data support a molecular basis underlying phenotypic variability in oligometastatic vs. polymetastatic disease. So far, the current body of knowledge considers that oligometastatic prostate cancer can be reasonably defined by up to five extrapelvic lesions.

## 3. Clinical Implications

From a clinical point of view, recent findings of the multi-arm STAMPEDE trial have fostered the approach of local radiotherapy of the primary tumor in de novo oligometastatic disease stages [26], while evidence for radical prostatectomy in this setting is still subpar [27]. 

Furthermore, evidence from randomized trials [28,29] has led to an increased use of metastasis-directed therapy (MDT) in oligorecurrent disease using stereotactic body radiotherapy (SBRT) in oligometastatic stages. In the ORIOLE phase 2 randomized clinical trial, 54 patients with omPCa, defined as up to three metastases based on conventional imaging and no ADT within the last 6 months, were randomized in a 2:1 fashion to undergo either observation or SBRT. At 6 months, biochemical, imaging or symptomatic progression was observed in 19% in the SBRT arm and in 61% in the observation arm. Regarding progression-free survival, the authors found a significant benefit for the SBRT arm (HR 0.31, 95% CI 0.13–0.75, *p* = 0.002). Remarkably, this benefit was reported only when all lesions were targeted. SBRT was generally well-tolerated and no grade 3 toxic effects were detected [28]. In the STOMP trial, patients with up to three extracranial metastases were randomly assigned to receive either metastasis-directed therapy (SBRT or surgery) of all detectable lesions or to undergo PSA surveillance. After a median follow-up of 3 years, the authors found a median ADT-free survival of 13 months for the surveillance cohort, compared to 21 months for the treatment cohort (HR 0.60, 80% CI 0.40–0.90, *p* = 0.11). The authors concluded that due to the prolonged ADT-free survival, metastasis-directed therapy should be further explored in phase 3 trials [30].

There is a paucity of data regarding local treatment in oligoprogessive disease. Only one small retrospective cohort study that included patients with metastatic castration-resistant prostate cancer reported the benefit of SBRT on median systemic treatment-free survival [31]. 

## 4. Biomarkers for the Identification of Patients with Truly Oligometastatic Prostate Cancer

While considerable advances have been achieved recently in imaging-based identification and classification of oligometastatic prostate cancer (omPCa), disease-specific biomarkers of clinical utility have not been established so far [32]. One of the challenges of molecular biomarkers is to identify patients with indolent disease, who will truly benefit from MDT alone, from patients with aggressive, high-risk polymetastatic disease, who may benefit from systemic therapy alone. Despite an unmet clinical need to implement characteristics of the tumor biology as a part of the omPCa distinction in order to only subject truly oligometastatic patients to metastasis-directed treatment options, preliminary findings have not been externally validated. Preferably, such biomarkers should be obtained in a non-invasive manner from body fluids, feasible for processing, reproducibility and affordability. Thus far, the most attracting options are represented by the detection of circulating tumor cells (CTCs), cell-free DNA or circulating tumor DNA (ctDNA) as well as miRNA, while liquid biopsies and next-generation sequencing are expected to play an increasingly important role in the clinical routine. A summary of results of current biomarker tests used the management of oligometastatic prostate cancer is presented in Table 2 [33].

### 4.1. Micro RNA 

Micro RNA (miRNAs) are short non-coding transcripts of 17–25 nucleotides involved in gene regulation at a post-transcriptional level. Different research groups were focused on the correlation between the differential expression of specific miRNAs and prostate cancer aggressiveness. Concerning the implication of miRNA in oligometastatic disease, it was demonstrated 10 years ago that oligometastatic patients present unique prioritized features of a microRNA classifier that includes the microRNA-200 family. In addition, the authors of this study created an oligometastatic–polymetastatic xenograft model in which the patient-derived microRNAs discriminated between the two metastatic outcomes, suggesting that the microRNA-200c enhancement in an oligometastatic cell line resulted in polymetastatic progression [37]. In addition, Uppal et al. analyzed the pathways targeted by microRNAs over-expressed in clinical oligometastasis samples, and found that miR-127-5p, miR-544a and miR-655-3p were encoded in the 14q32 microRNA cluster as co-regulators of multiple metastatic pathways through repression of shared target genes [38]. However, in a clinical trial, none of the miRNA targets presented a distinct expression between oligo- and polymetastatic prostate cancer patients [34].

Dhondt and collaborators aimed to find a stratification model that prospectively distinguished between oligo- and polymetastatic patients who progressed after primary treatment, combining clinicopathological characteristics and liquid biopsy (serum)-based miRNA expression profiles [34]. Notably, oligometastatic patients were allowed to have up to three metastases at baseline and not progress to more than three lesions following MDT or surveillance within 1 year of metastases diagnosis. In contrast, men with a polymetastatic disease revealed at least four metastases at baseline or developed at least four metastases within 1 year following imaging. The discovery and independent validation cohort consisted of 20 and 44 oligometastatic PCa, as well as 20 and 39 males with a polymetastatic PCa, respectively. In the discovery cohort, 14 miRNA candidates and PSA doubling time were demonstrated to be differentially expressed between oligo- and polymetastatic patients in the univariate analysis. The final prognostic model for oligometastatic disease was built on 10 predictors and was based on the discovery cohort reaching a promising predictive performance (AUC = 0.833). Unfortunately, none of the miRNA targets were differentially expressed between both groups in the validation cohort. Moreover, the multivariate model had no predictive ability in the validation set (AUC = 0.393).

### 4.2. Circulating Tumor Cells

Circulating tumor cells (CTCs) become detached from the primary tumor, migrate through the extracellular matrix and invade into vessels where they can be detected in samples of peripheral blood. The potential of CTC collection to serve as a prognostic biomarker in omPCa was recently demonstrated by Mandel et al. in the analysis of 33 patients from the ProMPT trial that assessed the role of cytoreductive radical prostatectomy in hormone-naïve omPCa (with up to three bone metastases) [35]. CTC enumeration in samples taken from males before and after surgery, carried out using the CellSearch system, revealed that patients with two or more CTCs/7.5 mL blood prior to surgery experienced a shorter time to castration-resistance and those with the same cut-off at 6 months after radical prostatectomy presented an inferior overall survival (OS). Regarding other malignancies, Hanssen and co-workers reported on a predictive value of CTCs in non-small cell lung cancer [39]. In particular, patients who were CTC positive (cut-off ≥1 CTC/7.5 mL blood) with oligo-brain disease (brain being the only metastatic site) exhibited a significantly worse overall survival. As opposed to these patients, it was proposed that patients who were CTC negative benefitted from intensified treatment, including the resection of both the primary tumor and brain metastases. Taken together, an omPCa classification, based on imaging as well as the negative status of CTC enumeration, could reveal the subset of males who may benefit the most from multimodal-aggressive treatment.

### 4.3. Immunophenotyping 

Immunophenotyping is the antibody-based assessment of specific antigen expression by a subset of cells aiming to identify their presence and estimate proportion in the whole cellular content. Immunophenotyping of peripheral blood mononuclear cells, isolated from 37 patients with omPCa (≤3 metastases) who were treated with metastasis-directed stereotactic body radiation therapy (SBRT) for all oligometastatic lesions, demonstrated that an increase in the number of tumor-reactive CD8^+^ T cells after intervention was associated with a diminished risk of disease progression in a study by Evans and co-workers [36]. At the same time, an increase in the central memory T cell subpopulation increased the risk of death, underscoring the potential of immune profiling for further biomarker investigations. Most recently, Zhang and collaborators described the outcomes of patients with oligometastatic castration-resistant PCa, identified by choline–PET/CT and treated with stereotactic ablative radiotherapy [40]. T cell immunophenotype analysis showed that high baseline levels of tumor-reactive T cells (TTR; CD8+CD11ahigh) predicted favorable local, biochemical and distant progression-free survival. In addition, an increase in TTR at day 14 from baseline was associated with prolonged overall survival. Thus, immunophenotyping merits further research activities targeting the evaluation of its predictive potential in an omPCa setting.

### 4.4. Circulating Tumor DNA

Circulating tumor DNA (ctDNA) originates from cancerous cells, and tumors and can be detected in the blood. The ORIOLE trial with 54 participants investigated the impact of stereotactic ablative radiotherapy (SABR) on disease progression in oligorecurrent disease [28]. Of note, no significant differences in ctDNA concentration were observed between men who did or did not experience disease progression in either arm. A high-risk mutation signature with truncating/pathogenic germline mutations was identified, while only males in a high-risk mutation-negative subgroup benefited from SABR. Despite that, the mutation analysis in this trial was limited to leukocyte DNA without being matched to tumor cells, allowing for the misinterpretation of some somatic mutations as being tumor-related, future research on the validation and expansion of these promising results in a marker-driven treatment decision is warranted. Strikingly, informative ctDNA analysis is possible even after neoadjuvant treatment. In patients treated with neoadjuvant chemotherapy before surgery for oligometastatic colorectal cancer, Pellini and co-authors demonstrated that molecular residual disease could be identified by the tumor-naive plasma ctDNA analysis [41]. Furthermore, oncogenomic analysis showed that four-fifths of patients might have been candidates for adjuvant immunotherapy based on a high ctDNA-inferred tumor mutational burden or targeted therapy based on actionable PIK3CA mutations. These encouraging findings underscore the potential of ctDNA analysis for individualized treatment and in omPCa disease in the future.

## 5. Impact of Molecular Imaging in Oligometastatic Prostate Cancer

An accurate assessment of a metastatic burden using different imaging techniques is crucial, especially in the absence of reliable molecular predictors. Current diagnostic tools such as computed tomography (CT), magnetic resonance imaging (MRI) and 99 m technetium–methylene–diphosphate (99mTc-MDP) bone scan present significant limitations, potentially leading to under-/overestimation of metastatic spread [42]. 

Positron emission tomography (PET) imaging presents excellent potential to measure metastatic burden, relying on differential tumor uptake and metabolism of various molecules such as glucose, acetate or choline.

Several radiotracers may be employed in the functional imaging of oligometastatic PCa, namely, 18 fluoro-2-D-deoxyglucose ([18F]-FDG), 11C-acetate [43], 11C-choline, 18F-choline, 18F-acetate [44], 18F-fluciclovine (also known as FACBC) [45], 68Ga-PSMA-11 [46], dihydrotestosterone-based radiotracers such as 16b-18F-fluoro-5a-dihydrotestosterone [47] and radiolabeled bombesin receptor antagonists that target gastrin-releasing peptide receptors [48], as well as other receptor-binding molecules (Table 3) [49,50,51].

18F-FDG represents the most frequently used tracer for functional imaging, but its role in PCa patients is limited due to the low FDG avidity of prostate cells in the absence of the “glycolytic switch” that is present in the majority of neoplasms [52]. However, FDG presents a high uptake in aggressive, poorly differentiated tumors such as small cell or neuroendocrine PCa, and can provide useful information in castrate-resistant adenocarcinoma [53].

**Table 3:** Overall detection rates of different PET–CT tracers in oligometastatic prostate cancer in different meta-analyses.

Tracer	De Novo		Oligo-Recurrent		Oligo-Progression	
	Sensitivity	Specificity	Sensitivity	Specificity	Sensitivity	Specificity
18F-FDG [54]	0.67; 95% CI: 0.55–0.77	0.72; 95% CI: 0.50–0.87	N/A	N/A	N/A	N/A
11C-acetate [54,55]	0.79; 95% CI: 0.70–0.86	0.82, 95% CI: 0.73–0.88	0.64 (0.59–0.69)	0.93 (0.83–0.98)	N/A	N/A
18F-fluciclovine [45]	0.57 (95% CI: 0.39–0.73)	0.99 (95% CI: 0.94–1.00)	0.68 (95% CI: 0.63–0.73)	0.68 (95% CI: 0.60–0.75)	N/A	N/A
11C/18F choline [56,57]	0.783; 95% CI, 0.718–0.836	0.792, 95% CI, 0.715–0.816	0.89 (0.80–0.94)	0.98 (0.95–0.99)	0.89 (0.80–0.94)	0.98 (0.95–0.99)
68Ga PSMA-11 [46,58,59]	0.97 (95% CI, 0.90–0.99)	0.66 (95% CI, 0.52–0.78)	0.93 (0.86–0.98)	0.96 (0.92–0.99)	0.76 (0.74–0.78)	0.45 (0.27–0.58)

The biological mechanisms for the accumulation of acetate are not yet fully understood, but several trials using C11-acetate PET imaging have generally demonstrated superiority in the detection of prostate cancer over FDG PET [43,60,61]. Acetate uptake in tumor cells is regulated by fatty acid synthase and acts as a probe of tissue metabolism through entry into catabolic or anabolic metabolic pathways, as mediated by acetyl-coenzyme A [61,62]. In patients with oligo recurrence and PSA levels <1 ng/mL, detection rates varied between 28.6 and 73.9% with a better uptake in the prostate bed and lymph nodes than 18F fluorocholine PET–CT [60,63]. However, due to a very short 20 min half-life and significant biologic heterogeneity within individual patient bone metastases, the usage of acetate in clinical practice has declined in the last decade [64,65].

Choline acts as a precursor for the biosynthesis of phospholipids, e.g., phosphatidylcholine, which are involved in the function of cell membranes and also in the modulation of cellular signaling responsible for cell proliferation and transformation [66]. Choline, labelled with 11C or 18F, represents the most frequently used tracer in the last decade to investigate biochemical failures after local treatment for PCa [44,66,67]. Although both tracers share the same indications, 11C-choline presents a lower rate of radioactive urine in the bladder than 18F, making it a better candidate for exploration of the prostate/prostate bed. The rate of detection of PCa by 11C/18F choline PET/CT is similar across different meta-analyses, with a pooled sensitivity varying between 85 and 89% and a pooled specificity between 87 and 92.6% [56,68,69,70]. 

The role of choline PET in oligometastatic recurrent disease has been demonstrated by several prospective trials performed in order to select patients suitable for salvage therapy, including metastasis directed therapy (MDT) [27,30,71]. Although MDT in patients with nodal oligo-recurrence improves cancers-specific survival when compared with the standard of care, the current guidelines do not strongly suggest the use of routine choline PET in the evaluation of BCR, except for cases with PSA >1 ng/mL and where there are no Ga-PSMA-PET CT available [67].

18F-fluciclovine (anti-1-amino-3-18F-fluorocyclobutane-1-carboxylic acid (18F-FACBC)) was approved by the FDA in May 2016 in the setting of early-recurrent PCa [72]. This molecular tracer targets the amino acid transporters ASCT2 and LAT1 on cell surfaces which are overexpressed in prostate cancer [73]. In 2016, Nanni et al. reported that the overall detection rates of 18F FACBC PET–CT were higher compared with choline PET–CT regarding local, lymph nodal and bone relapse. The authors, additionally, have shown a higher sensitivity (37% vs. 32%) and specificity (67% vs. 40%) in the setting of patients with biochemical relapse after radical prostatectomy [74]. However, in the LOCATE trial, which evaluated 213 patients with BCR after local treatment from a 15 center US study, the authors reported that 18F-fluciclovine PET/CT disease detection rates may differ significantly among centers, particularly at PSA levels ≤1.0 ng/mL, mainly due to readers’ training and experience in PET scan interpretation [29]. Several papers have shown various degrees of physiologic uptake in healthy tissues, including the liver, bone marrow, lung, myocardium, pancreas, pituitary and salivary glands, bowel and muscles. Additionally, 18F-fluciclovine image interpretation is predominantly visual/qualitative, unlike other radiotracer image interpretation, which often rely more on semiquantitative standardized uptake values (SUVs) [75,76]. The diagnostic accuracy of 18F-fluciclovine PET was compared with that of 68Ga-PSMA-11 PET in a prospective, single-center, single-arm comparative trial in patients with HSPC that were candidates for salvage therapy for first-time BCR and PSA less than 2.0 ng/mL (NCT03515577) [77]. The detection rates were significantly lower with 18F-fluciclovine PET (13/50 (26%; 95% (CI) 15–40)) than with 68Ga-PSMA-11 PET (28/50 (56%; 41–70)), with a HR of 4.8 (95%CI 1.6–19.2; *p* < 0.0026). 

PSMA, also known as glutamate carboxypeptidase II, N-acetyl-a-linked acidic dipeptidase I or folate hydrolase, is a type II transmembrane glycoprotein belonging to the M28 peptidase family [78]. PSMA is located in the cytosol in normal prostate cells and acts as a glutamate carboxypeptidase on various substrates, including the nutrient folate and the neuropeptide N-acetyl-L-aspartyl-L-glutamate [79]. However, PSMA is not specific to prostate tissue. Significant PSMA uptake can be present in various other tissues, such as brain, kidney, salivary glands, liver, ganglia and small intestine [80].

A decade ago, PSMA was first used for imaging of PCa using SPECT as a receptor ligand for 111 In-capromab pendetide (Prostascint) [81]. However, the images were technically challenging to read and expensive to perform since Prostascint targets the intracellular domain of PSMA with a long half-life and long blood-pool activity, resulting in inferior image quality [63]. 68Ga-PSMA-11 (also known as HBED-CC, Glu-urea-Lys(Ahx)-HBED-CC and PSMA-HBED-CC) was introduced as a molecular tracer for PET CT that targets the extracellular domain of PSMA [78]. The imaging technique 68Ga PSMA-11 PET–CT appears to be superior to PET–CT with other tracers, demonstrating increased avidity of uptake and a favorable lesion-to-background ratio, especially at low PSA levels [82]. 

The utility of 68Ga PSMA-11 PET–CT in a de novo metastatic setting has been recently evaluated in a meta-analysis [46]. On a per-patient analysis, the sensitivity and specificity of 68Ga-PSMA PET were 77% and 97%, respectively, after RP and pelvic lymph node dissection. On a per-lesion analysis, the sensitivity and specificity were 75% and 99%, respectively. Despite the findings of this meta-analysis, the authors state that there is clearly a need for more robust data from prospective trials, since the majority of studies included were retrospective and single-center experiences.

In the oligo recurrent setting, the role of 68Ga PSMA-11 PET–CT has been demonstrated in several meta-analysis, with higher percentages of positive results for metastases in lymph nodes and bone, particularly at low pre-PET PSA levels, that varied between 33–95% [46,58,83]. Based on the available data, 68Ga-PSMA PET seems to have a high management impact in patients considered for SRT at (early) BCR after RP [84]. Despite the high positive detection rate, the impact 68Ga PSMA-11 PET–CT on the clinical management of omPCa patients is yet to be demonstrated since the majority of studies published are retrospective, with a small number of patients included and with significant heterogeneity of adjuvant treatments [80].

The incidence of oligometastatic disease will likely increase in the future due to the availability of advanced molecular imaging. However, one patient staged oligometastatic with one tracer might consider polymetastatic using another tracer. It is mandatory to take into account the clinical history of the patient in order to interpret the imaging results and decide whether a change in treatment is required. 

The future is certainly a combination of various tracers that target different metastatic sites. The EORTC imaging group suggested a clinical algorithm to integrate modern imaging methods into care pathways to identify oligometastatic disease in PCa scenarios [71]. Recently, Eiber et al. proposed a molecular imaging tumor, node and metastasis system (miTNM Version 1.0) as a standardized reporting framework for PSMA–ligand PET/CT or PET/MRI [85]. However, these systems need to be validated in future clinical trials.

## 6. Conclusions

There is an urgent unmet clinical need to implement characteristics of tumor biology as a part of oligometastatic prostate cancer diagnosis in order to increase the results of metastatic directed therapy. Thus far, the research regarding genetic, epigenetic and immune-related pathways is still unable to distinguish between an oligo- and polymetastatic state. In the absence of reliable molecular predictors, PET–CT remains the only available tool to quantify the metastatic burden. 68Ga PSMA-11 PET–CT shows promising results, but there is clearly a need for more robust data from prospective trials.

## Figures and Tables

**Table 1:** Results of epigenetic drugs in clinical trials for prostate cancer. (mCRPC—metastastic castrate-resistant prostate cancer, rPF—radiographic progression-free, OS—Overall survival, mPC—metastatic prostate cancer, PFS—progression-free survival).

Drug Name	Target	Combination	Phase	Indication	Identifier	Results
Panobinostat [11]	Histone deacetylases (HDACs)	Panobinostat 40 mg (arm A) or 20 mg (Arm Bin combination with bicalutamide 50 mg/day in 3-week cycles	II	mCRPC pts. resistant to second-line antiandrogen therapy	NCT00878436	% of pts with rPF—47.5% in arm A 38.5% in arm B
Vorinostat/ suberoylanilide hydroxamic acid (SAHA) [12]	Histone deacetylases (HDACs)	400 mg vorinostat/SAHA orally each day	II	mCRPC with disease progression on prior chemotherapy	NCT00330161	Median time to progression—2.8 months OS—11.7 months
Azacitidine [13]	DNA methyltransferases (DNMT)	Azacitidine + docetaxel + prednisone 5 mg	I/II	mCRPC pts. who progressed during or within 6 months of docetaxel	NCT00503984	PSA response >50% in 52.6% of pts. Radiographic response—30% of pts
5-Aza-2-deoxycytidine (decitabine) [14]	DNA methyltransferases (DNMT)	Decitabine 75 mg/m^2^/dose	II	recurrent mPC after total androgen supression		Stable disease—16.66% of pts at 10 months
ZEN-3694 [15]	Bromodomain and extraterminal (BET)	ZEN-3694 plus enzalutamide	Ib/IIa	progressive mCRPC with prior resistance to abiraterone and/or enzalutamide	NCT02711956	Median rPFS—9 monthsPFS—5.5 months
GSK525762 [16]	Bromodomain extraterminal (BET)	GSK525762+ Abiraterone/prednisone or enzalutamide	1	mCRPC with disease progression on prior chemotherapy	NCT03150056	Active, not recruiting

**Table 2:** Results of current biomarker tests in oligometastatic prostate cancer state.

Test	Population	No of pts.	Intervention	Prognostic Performances	Comments
Somatic next-generation sequencing (NGS) [17]	Metastatic castrate sensitive or biochemically recurrent	45—Oligorecurrent 102—Oligometastatic 22—Metachronous polymetastatic 125—De novo metastatic	Foundation one CDx (324—gene panel) and personal genome diagnostics cancer SELECT 125 (125—gene panel) assays	TP53 and WNT pathway genes can predict patterns of metastatic dissemination	Significant heterogeneity in imaging, treatment and oncological endpoints
MicroRNA gene expression [34]	Oligo- and polymetastatic recurrent diseasese	20 Polymetastatic and 20 with oligometastatic disease	miRNA expression profiles using 41 miRNA targets	Sensitivity 0.894 (0.714–1.000) Specificity 0.492 (0.203–0.782)	No predictive ability in multivariate model
Circulating tumor cells (CTCs) [35]	Hormone-naïve oligometastatic prostate cancer	33 patients with bone metastasis and prostate cancer	CTC enumerations before and after cytoreductive radical prostatectomy	2 or more CTCs/7.5 mL blood prior to surgery experienced a shorter time to castration-resistance	Probability was assessed using Harrell’s C concordance measurement
CD8+ T-cell subpopulations [36]	Castrate-resistant oligo recurrent	37 patients who progressed after primary treatment	Metastasis-directed stereotactic body radiation therapy	Increase in the TCM cell subpopulation was associated with the risk of death (HR, 1.22 [95% CI, 1.02–1.47],	Significant heterogeneity in the recurrent omPCa patient population
Circulating tumor DNA (ctDNA) [28]	Oligo recurrent	54 patients who progressed after primary treatment	Metastasis-directed stereotactic body radiation therapy	Increased peripheral baseline clonality was associated with progression at 180 days in SABR arm	No association between baseline ctDNA concentration and oncological outcomes
